# A ‘sea anemone’ onychomatricoma

**DOI:** 10.1016/j.jpra.2021.05.009

**Published:** 2021-05-28

**Authors:** Xi Ming Zhu, Waleed AlBadry, Andrew Fleming

**Affiliations:** a St. George's University Hospitals NHS Foundation Trust, Tooting, Blackshaw Road, Tooting, London SW170QT, United Kingdom; b St. George's, University of London, United Kingdom; c Department of Plastic Surgery, St. George's University Hospitals, NHS Foundation Trust, Tooting, London, United Kingdom; d Department of Plastic Surgery, Cairo University, Egypt

**Keywords:** Nail bed tumour, Onychomatricoma, Hand surgery, Plastic surgery

## Abstract

**Objective:**

Here we describe the presentation, identification, operation details and subsequent histological analysis of an onychomatricoma, a benign rare subungual tumour that is often misidentified and diagnosed.

**Methods:**

No public involvement to declare, patient was consented for surgical excision and provided verbal consent to photography and its use in publishing.

**Participant:**

One 44-year-old female.

**Intervention:**

One time surgical excision.

**Primary and secondary outcomes measured:**

Not applicable.

**Results:**

Complete excision and histopathological report of specimen provided.

**Conclusion:**

Complete surgical excision is an effective means of treatment for onychomatricoma.

## Strengths and limitations

A 44-year-old right-handed female was referred for a dystrophic nail affecting her left middle finger for the past four years. There was no associated pain or loss of function. The patient was on regular fluoxetine and was otherwise fit and well. Examination revealed yellow discolouration and a subtle fullness of the medial nail plate on the left middle finger.

Initial working differential diagnoses included myxoid cyst, ganglion, or glomus tumour. MRI of the left hand detected a small, ovoid, enhancing 2 × 1 mm lesion with no underlying bony erosion, suggestive of a small glomus tumour.

Operatively, the nail plate was removed, revealing a peculiar frond-like tumour occupying the germinal matrix. The tumour had invaded the proximal soft tissues locally and was completely excised in continuity.

Histological examination of the 4 × 3 mm specimen on microscopy showed an exophytic fibroepithelial tumour formed from tall and broad papillary structures covered by a thin layer of matrical type epithelium. The core of the papillary structures consisted of uniform fibroblast-like cells with collagen fibres arranged in a vertical orientation. A few capillaries were noted within the stroma. The overall configuration resembled that of a sea anemone.

Sections of the deep edge of the nail plate showed jigsaw-like variations, likely due to invasion by the finger-like projections of the tumour. Immunohistochemistry revealed background stromal staining with CD34, with negative staining of: S100, actin, desmin, EMA, AE1/3, CK8/18.

**Fig. 1:**
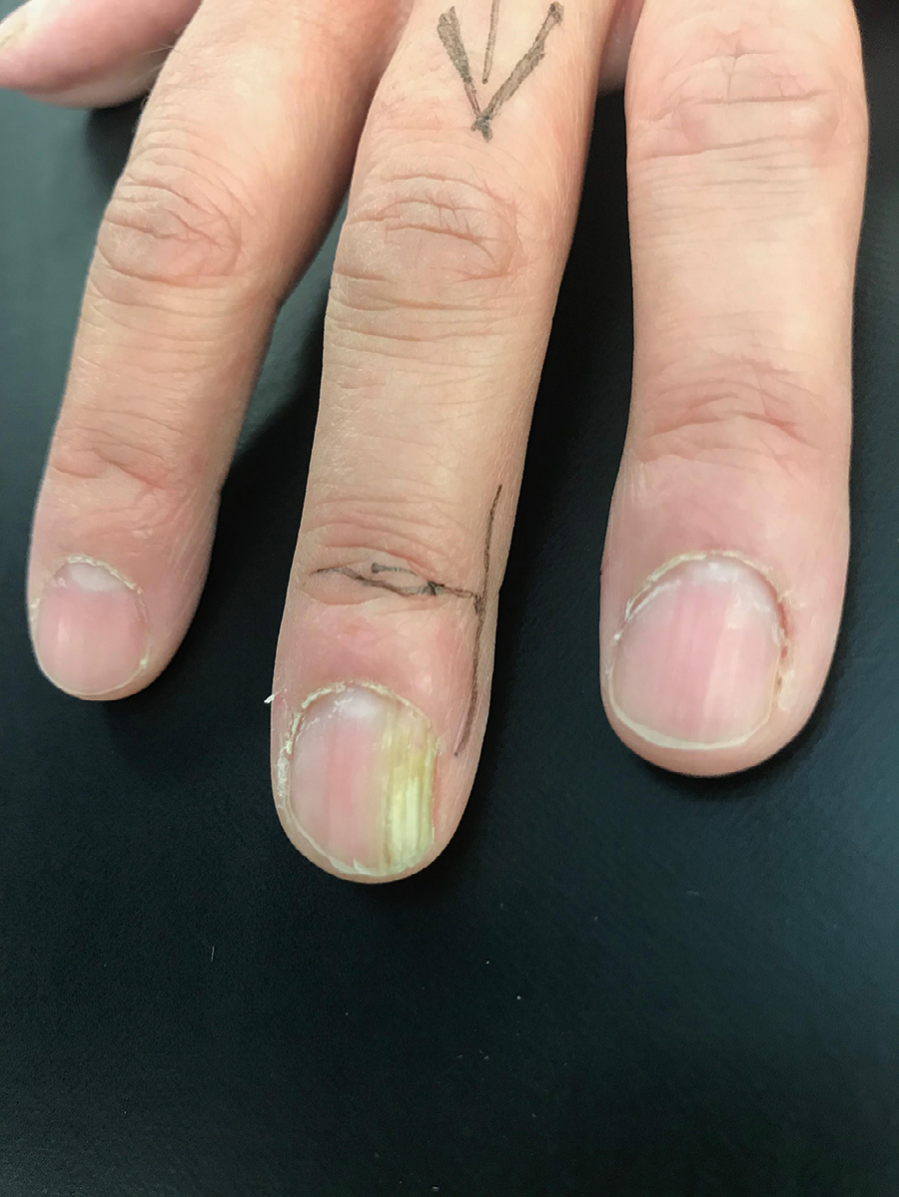
Pre-operative appearance of the nail.

**Fig. 2:**
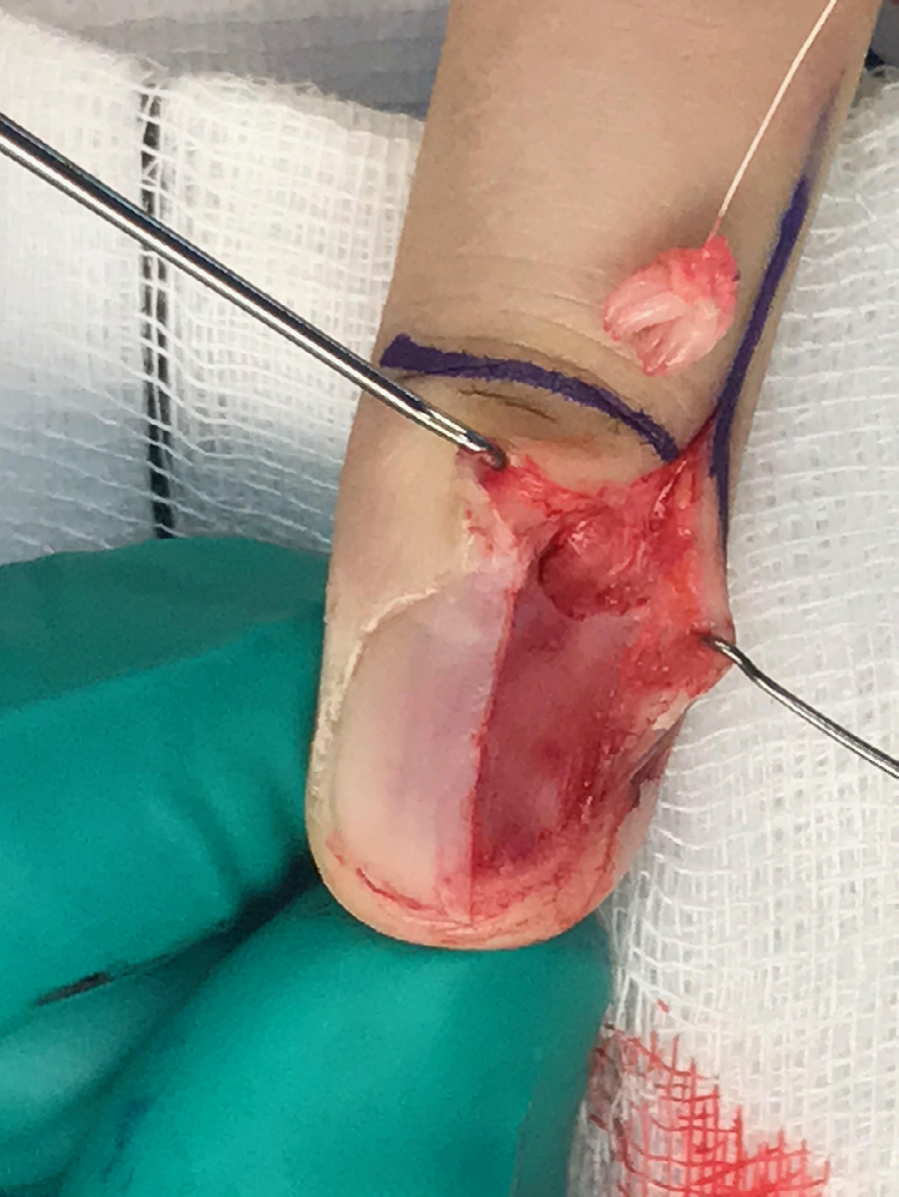
Removal of the subungual sea-anemone shaped onychomatricoma.

No dysplasia or malignancy was identified, and the overall picture favoured an onychomatricoma. The operative site has healed well and there has been no recurrence at latest follow-up. Figs. 1,2

## Discussion

Onychomatricoma is a rare benign subungual tumour affecting the germinal nail matrix, first reported by Baran and Kint in 1992.^1^ The tumour classically presents with a tetrad of: yellow discolouration with thickening of the nail plate, splinter haemorrhage, transverse nail plate overcurvature, and worm-like cavitation of the nail plate.^2^ The presence of frond-like cavitations in the nail plate is further suggestive of onychomatricoma.^2^

Differentials for onychomatricoma include Bowen's disease, fibrokeratoma, onychomycosis, squamous cell carcinoma, and subungual verruca vulgaris amongst others.^3^ Nevertheless, onychomatricoma appears to be the only one that actively produces nail plate substance, lending to its thickening. Where doubt remains, diagnosis can be supported by microscopy, immunohistochemistry, and imaging.

Complete excision remains the treatment modality of choice.

## Statements

 

## Declaration of Competing Interest

We have no competing interests to declare.
